# The Woman Worker's World

**Published:** 1918-08-10

**Authors:** 


### THE WOMAN WORKER'S WORLD.

The more thriftless of our working women waste infinite numbers of hours in idle-handed gossip, clothing their children in too numerous layers of ragged and not-too-clean garments because they do not know how to set to work to make, remake, or repair. The great lesson needed is construction, and the great point of this teaching is to show how from the same pattern a number of different garments can be cut with slight adaptation. The best parts of two grey woollen vests can be used to form a child's "Princess" petticoat, bordered with blanket stitch in contrasting colour, a white serge skirt can become a child's tunic and trousers, or knickers. That three layers of clothing are enough for a child, plus outdoor coat when needed, is another of the most important lessons in domestic hygiene.

Some nurses or probationers who wish to combine missionary with medical work may be glad to know of the classes for women held at King's College, Strand, which will be continued in the Michaelmas term, probably on Saturday mornings. There will be a preliminary class for beginners, and two courses, (A) St. Mark's Gospel, (B) Text and Theology of the Epistles to Philemon, Colossians, and Ephesians. Information as to syllabus, dates, and fees may be obtained from Miss G. M. Ireland Blackburne, 14 Motcomb Street, S.W. 1, or at the College before term begins.

A strong committee, with the Rev. J. C. Pringle, of the C.O.S., as chairman, has been formed to establish a hostel for the reception of girls and women who are undergoing treatment in London for venereal disease. The movement is most important, for patients who are not of the poorest class, and who have only to attend for a few hours at a time for hospital treatment, may easily be placed in a great dilemma, so much so that the temptation to throw up the cure may be very strong. It is proposed, if possible, to open another hostel where mothers with infants may be received. Meantime £2,000 is required to carry on the one now opened, and subscriptions will gladly be received by Mr. T. Slingsby Tanner at 48 Lower Sloane Street, S.W. When necessary, work is sought for the inmates, and training arranged if desirable. The title "After-care Hostel" is therefore used. Information may be obtained from Miss Orred, 59 Lowndes Square, S.W.

An extraordinarily interesting exhibition of home-made toys is being shown at Cambridge during the University Extension fortnight. Some of the models of wooden furniture with raffia seats, of dolls' size, have been sent to Holland for the occupations of interned prisoners. Others have occupied the leisure of Belgian children. All show how wonderfully contrivance can develop intelligence. For example, a milliner's shop, nominally that of Messrs. Mears and Swells, and offering to make up ladies' own minds while they wait, is most dexterously contrived with furniture of match-boxes, and stands of photographic reels surmounted by millinery contrived from every imaginable remnant. Pill-boxes become frames for highly fashionable toques. In a model flower shop they become plant pots; corks coloured red serve the same purpose. A communal kitchen, a professor's study, an Oriental bazaar, and many more delightful toys are contrived from waste products on these lines. It is hoped that the show will be seen in London later on, and prove valuably suggestive to teachers and to managers of children's hospitals and convalescent homes, amongst others.

